# Genetic parameters and genomic prediction of growth and breast morphological traits in a crossbreed duck population

**DOI:** 10.1111/eva.13638

**Published:** 2024-02-07

**Authors:** Wentao Cai, Jian Hu, Wenlei Fan, Yaxi Xu, Jing Tang, Ming Xie, Yunsheng Zhang, Zhanbao Guo, Zhengkui Zhou, Shuisheng Hou

**Affiliations:** ^1^ Institute of Animal Science Chinese Academy of Agricultural Sciences Beijing China; ^2^ College of Animal Science and Technology Qingdao Agricultural University Qingdao China; ^3^ College of Animal Science and Technology Beijing University of Agriculture Beijing China

**Keywords:** Bayesian models, genetic correlation, genomic selection, heritability, single‐step

## Abstract

Genomic selection (GS) has great potential to increase genetic gain in poultry breeding. However, the performance of genomic prediction in duck growth and breast morphological (BM) traits remains largely unknown. The objective of this study was to evaluate the benefits of genomic prediction for duck growth and BM traits using methods such as GBLUP, single‐step GBLUP, Bayesian models, and different marker densities. This study collected phenotypic data for 14 growth and BM traits in a crossbreed population of 1893 Pekin duck × mallard, which included 941 genotyped ducks. The estimation of genetic parameters indicated high heritabilities for body weight (0.54–0.72), whereas moderate‐to‐high heritabilities for average daily gain (0.21–0.57) traits. The heritabilities of BM traits ranged from low to moderate (0.18–0.39). The prediction ability of GS on growth and BM traits increased by 7.6% on average compared to the pedigree‐based BLUP method. The single‐step GBLUP outperformed GBLUP in most traits with an average of 0.3% higher reliability in our study. Most of the Bayesian models had better performance on predictive reliability, except for BayesR. BayesN emerged as the top‐performing model for genomic prediction of both growth and BM traits, exhibiting an average increase in reliability of 3.0% compared to GBLUP. The permutation studies revealed that 50 K markers had achieved ideal prediction reliability, while 3 K markers still achieved 90.8% predictive capability would further reduce the cost for duck growth and BM traits. This study provides promising evidence for the application of GS in improving duck growth and BM traits. Our findings offer some useful strategies for optimizing the predictive ability of GS in growth and BM traits and provide theoretical foundations for designing a low‐density panel in ducks.

## 1 INTRODUCTION

Domestic ducks are valuable poultry with a sizable global consumer base in various parts of the world, especially in Asia and Europe. Duck meat is generally considered flavorful, abundant in amino acids and polyunsaturated fatty acids, and relatively low in fat content. The production and consumption of duck meat significantly have been increasing around the world, particularly in the case of China. The most important duck traits, according to breeders, are related to body weight composition (e.g., weight gain and breast muscle thickness (BMT)) (Szwaczkowski et al., 2010). The duck industry's profitability relies on increasing the growth and proportion of key carcass segments, particularly the breast muscle (He et al., 2011). However, in spite of the importance of these traits, relatively little is known about the genetic improvement of growth and breast morphological (BM) traits using genomic selection (GS). GS can predict the genetic and phenotypic values of a genotyped individual by using genotypes and phenotypes from a reference population, which was first put forth by Meuwissen et al. (2001). GS utilizes both genomic and pedigree information to estimate breeding value, which can be more accurate than the method using only pedigree information (Meuwissen & Goddard, 2010; VanRaden et al., 2009), usually called “best linear unbiased prediction” (BLUP) (Henderson, 1975). GS can also shorten the generation interval by selecting individuals at their early life (Boichard et al., 2016). Over the past decade, GS is an effective and widely used approach to accelerate genetic improvement and reduce breeding cost in livestock (VanRaden et al., 2009), poultry (Liu et al., 2014; Tan et al., 2022), and aquatic animals (Joshi et al., 2021). For ducks, estimates of prediction accuracy have only been reported for carcass and meat quality traits (Cai et al., 2023; Zhang et al., 2022).

GS prediction accuracy is affected by the choice of statistical models. Since genomic BLUP (GBLUP) is easier to implement and less computationally demanding, it is widely used in routine genomic evaluation (VanRaden, 2008). Compared to GBLUP model, the single‐step genomic BLUP (ssGBLUP) model has the advantage in taking advantage of phenotype and pedigree records for ungenotyped individuals and obtaining genetic merits of both genotyped and ungenotyped individuals in one equation (Legarra et al., 2014; Misztal et al., 2013), which helped increase GS accuracy in various studies (Gao et al., 2019; Yan et al., 2018). In terms of modeling the distribution of marker effects, Bayesian models are superior to GBLUP (Meuwissen et al., 2001), which can improve the accuracy of GS (Lopes et al., 2021; Shi et al., 2021). The density of single‐nucleotide polymorphism (SNP) markers also has a noticeable impact on the prediction accuracy of GS (Krishnappa et al., 2021). The prediction accuracy would not be improved when the marker density reached a certain degree (Wang et al., 2017, 2018). In contrast, breeding costs and computational time would be dramatically increased. As a duck commercial SNP array has not been developed, it remains unclear what the optimal marker density is for duck GS, such as when accuracy reaches a plateau.

Nowadays, duck breeding is mainly conducted through conventional phenotypic selection and pedigree followed by the evaluation of estimated breeding values (EBVs), using BLUP strategy. Until now, there has been limited research conducted on the application of genomic prediction in ducks. The only two reports on duck GS focused on meat quality (Zhang et al., 2022) and carcass traits (Cai et al., 2023). However, the use of genomic prediction has never been investigated in duck growth and BM traits. Therefore, it is desirable to estimate predictive reliability gains of these economic traits from using genomic evaluations instead of traditional BLUP evaluations.

Thus, the objective of this study was to calculate genetic parameters of body weight (BW), average daily gain (ADG), and BM traits at a crossbreed duck population and compare the predictive reliabilities of their breeding values using pedigree BLUP, GBLUP, ssGBLUP, and Bayesian models. Additionally, how the density of markers affects predictive reliabilities of GS was reported.

## 2 MATERIALS AND METHODS

### 2.1 Population data

In this study, an experimental cross‐population of Pekin duck × mallard consists of 1893 individuals was analyzed. The phenotypes of four BW traits were weighted at 1 day (BW_1_), 19 days (BW_19_), 28 days (BW_28_), and 56 days (BW_56_). These five ADG traits were calculated using weight difference during 1 and 19 days (ADG_1–19_), 1 and 28 days (ADG_1–28_), 1 and 56 days (ADG_1–56_), 19 and 56 days (ADG_19–56_), and 28 and 56 days (ADG_28–56_). The phenotypes of four BM traits were measured in vivo at the age of 56 days. Keel length (KL) was the distance between the front and rear ends of the keel measured by a caliper, and the breast muscle width (BMW) was the distance between the two shoulder joints measured by a caliper. BMT and skin fat thickness (SFT) were measured at the middle of keel and 2 cm to the left of the border of keel by a portable B‐ultrasound apparatus. The breast muscle volume (BMV) was calculated using KL × BMW × BMT. Table 1 describes the number of animals with observations with mean, standard deviation (SD), and minimum and maximum values for each trait. A total of 941 ducks were genotyped using resequencing technology on an Illumina HiSeq X Ten with an average ×5 coverage, which were described in our previous study (Zhou et al., 2018). We aligned all the 150‐bp paired‐end clean reads to the Pekin duck reference genome (GCA_003850225.1) using BWA software (v0.7.17) (Li & Durbin, 2009). The alignment quality was improved by Picard (v2.24.1) (Broad Institute, 2019). The SNP calling was conducted by GATK HaplotypeCaller module (v3.5) (Van der Auwera et al., 2013). We removed the SNPs with minor allele frequency (MAF) <0.05, call rate <90%, and *p*‐value of Hardy–Weinberg equilibrium test <10^−6^ using PLINK (v1.90) (Purcell et al., 2007). The SNPs located in non‐autosome were also removed. Finally, a total of 1,037,662 SNPs for 941 individuals were obtained for genomic prediction.

**TABLE 1:** Phenotype description of the Pekin duck × mallard F2 population.

Category	Traits^a^	N_obs^b^	N_gen^c^	Mean	SD	Max	Min	Heritability
Body weight (g)	BW_1_	1893	941	45.7	5.0	63.2	25.4	0.72 ± 0.20
	BW_19_	503	326	680.1	77.0	966.0	401.0	0.62 ± 0.16
	BW_28_	536	461	1149.4	151.8	1653.0	438.0	0.57 ± 0.14
	BW_56_	1170	935	1894.7	262.1	2859.0	951.0	0.54 ± 0.08
Growth (g/day)	ADG_1–19_	503	326	33.4	4.0	48.4	19.1	0.57 ± 0.16
	ADG_1–28_	536	461	39.4	5.4	57.7	14.3	0.56 ± 0.14
	ADG_1–56_	1170	935	33.0	4.7	50.2	16.4	0.53 ± 0.08
	ADG_19–56_	494	325	31.1	5.5	48.2	8.4	0.21 ± 0.12
	ADG_28–56_	513	457	27.6	6.4	51.7	4.2	0.24 ± 0.15
Breast morphological (cm or cm^3^)	KL	1168	933	12.0	0.7	14.0	9.0	0.33 ± 0.07
	BMW	1168	933	9.6	0.5	11.1	5.2	0.29 ± 0.06
	BMT	1163	935	1.1	0.1	1.5	0.4	0.33 ± 0.07
	BMV	1161	933	121.9	23.6	208.4	34.6	0.39 ± 0.07
	SFT	1162	935	0.4	0.1	0.8	0.1	0.18 ± 0.05

Abbreviations: BMT, breast muscle thickness; BMV, breast muscle volume; BMW, breast muscle width; KL, keel length; SFT, skin and fat thickness.

^a^ BW_1_ = body weight (1 day); BW_19_ = body weight (19 days); BW_28_ = body weight (28 days); BW_56_ = body weight (56 days); ADG_1–19_ = average daily gain from 1 day to 19 days; ADG_1–28_ = average daily gain from 1 day to 28 days; ADG_1–56_ = average daily gain from 1 day to 56 days; ADG_19–56_ = average daily gain from 19 days to 56 days; ADG_28–56_ = average daily gain from 28 days to 56 days.

^b^ N_obs = numbers of individuals with phenotypic records.

^c^ N_gen = numbers of genotyped individuals.

### 2.2 BLUP, GBLUP, and ssGBLUP models

The following linear mixed model was used to estimate breeding values using pedigree and phenotype information for BLUP (Henderson, 1975), genotype and phenotype information for GBLUP (VanRaden, 2008), and pedigree, genotype, and phenotype information for ssGBLUP (Legarra et al., 2009):

y=Xβ+Za+e,

where ***y*** was the vector of observation; ***β*** was the vector of fixed effects, including sex and feed room; ***X*** was incidence matrix for fixed effects; ***a*** was the vector of additive genetic effects; ***Z*** was incidence matrix to allocate phenotypic observations to individuals; and ***e*** was random residual effects.

In BLUP, ***a*** was the vector of additive genetic effects and assumed that ***a*** ∼ *N*(0, ***A*** $\sigma_{a}^{2}$), in which ***A*** was the numerator relationship matrix obtained from the pedigree, $\sigma_{a}^{2}$ was additive genetic variance, and $\sigma_{e}^{2}$ was error variance. Heritability ($h^{2}$) was calculated by $\sigma_{a}^{2}$ / $\;\left(\sigma_{a}^{2}{+\sigma }_{e}^{2}\right)$. The heritabilities were defined as follows: >0.4 high; 0.2–0.4 moderate; and <0.2 low (Santana et al., 2020). The correlation coefficient values were interpreted as follows: 0.0–0.2 little; 0.2–0.4 weak; 0.4–0.7 moderate; and 0.7–1.0 strong (Guilford, 1950).

In GBLUP, ***a*** ∼ *N*(0, ***G*** $\sigma_{a}^{2}$), in which genomic relationship matrix ***G*** was calculated by VanRaden's method (VanRaden et al., 2009), the formula was $G=\frac{{\mathrm{ZZ}}^{\prime }}{2\sum p_{i}\left(1-p_{i}\right)}$, where ***Z*** was the SNP markers’ incidence matrix, and it is the genotype matrix (***M***, 0 1 2) minus the mean of marker across individuals 2$p_{i}$, ***Z*** = ***M*** −2$p_{i}$, where $p_{i}$ is the MAF at each SNP.

In ssGBLUP, ***a*** ∼ *N*(0, ***H*** $\sigma_{a}^{2}$), in which ***H*** was blended relatedness matrix combines pedigree and the genetic markers. The inverse of ***H*** was derived given ***A*** and ***G*** via the following formula:

$$H^{-1}=A^{-1}+\left[\begin{array}{cc} 0 & 0\\ 0 & G^{-1}-A_{22}^{-1} \end{array}\right]$$

where ***A***_**22**_ was a numerator relationship matrix for genotyped animals, ***A*** was a pedigree relationship matrix, and ***G*** was a genomic relationship matrix.

Variance components and EBVs or genomic estimated breeding values (GEBVs) were estimated by restricted maximum likelihood (REML) implemented in the ASReml‐R (v4.2) (Butler et al., 2017).

### 2.3 Bayesian models

We used five Bayesian models with different assumed distributions of SNP effects. The model was given as follows:

Y=Xβ+Zs+e.

where ***y***, ***X***, ***β***, ***Z***, and ***e*** were the same as terms described in the above model, and s was the sum of the vector of SNP effects derived from different assumed distributions. Here, BayesB assumed that SNP effects had a mixture prior, where most of the genetic markers have zero effect with probability 1 − *π*, and seldom markers obeyed a scaled *t*‐distribution with probability *π* (Meuwissen et al., 2001). BayesCπ assumed that SNP effects had a mixture prior to a normal distribution that had mean 0 and variance *σ* ^2^ with probability *π* and null‐effect markers with probability 1 − *π* (Habier et al., 2011). BayesN and BayesS were modified models based on BayesCπ. BayesN is a nested mixture model, accounting for the dependence of effects of SNPs that are closely linked, where the SNPs within a 0.2 Mb non‐overlapping genomic region are collectively considered as a window. BayesS assumed the variance of non‐zero SNP effects was affected by MAF ($p_{i}$) through a parameter *S* ($\sigma_{i}^{2}={\left[2p_{i}\left(1-p_{i}\right)\right]}^{S}\sigma^{2}$) (Zeng, de Vlaming, et al., 2018). BayesR assumed that SNP effects had a mixture of four normal distributions *N*(0,$\;\gamma_{k}\sigma_{k}^{2}$); the$\;\gamma_{k}$ were 0, 0.01, 0.1, and 1 with probability $\pi_{1}$, $\pi_{2}$, $\pi_{3,}$ and $\pi_{4}$, respectively ($\pi_{1}+\pi_{2}+\pi_{3}+\pi_{4}=1$) (Moser et al., 2015). GCTB (V2.01) was used to calculate unknown parameters and SNP effects of Bayesian models using a Gibbs scheme based on Markov chain Monte Carlo (MCMC) iterations (Zeng, de Vlaming, et al., 2018). A total of 21,000 MCMC iterations were generated, with the first 1000 iterations discarded as burn‐in and every 10th sampled values saved for inferring posterior statistics.

### 2.4 Cross‐validation and permutation of marker densities

The prediction reliability of the models was estimated based on a fivefold cross‐validation. We randomly divided these genotyped animals with phenotypes into five subsets. Each subset at a time was predicted using the remaining four subsets, which means the number of genotyped animals in training and validation populations was 753 and 188, respectively. After the EBVs/GEBVs for each validated animal were predicted using the above models, we calculated the square of Pearson's correlation coefficient between predicted EBVs/GEBVs and adjusted phenotypic values in validation population. We defined the prediction reliability using the average of the square of Pearson's correlation coefficients based on a fivefold cross‐validation.

To assess the effect of marker density on the performance of GS, we randomly selected 0.5, 1, 3, 5, 10, 50, 100, and 500 K from the original 1.04 million (M) markers. Each selected marker set was used to build the genomic relationship matrix and calculate GEBVs using the GBLUP model. The prediction reliability of the models was estimated based on a fivefold cross‐validation (see above). For each density of marker, we repeated this process 30 times to obtain average results.

## 3 RESULTS

### 3.1 Genetic parameters

**FIGURE 1:**
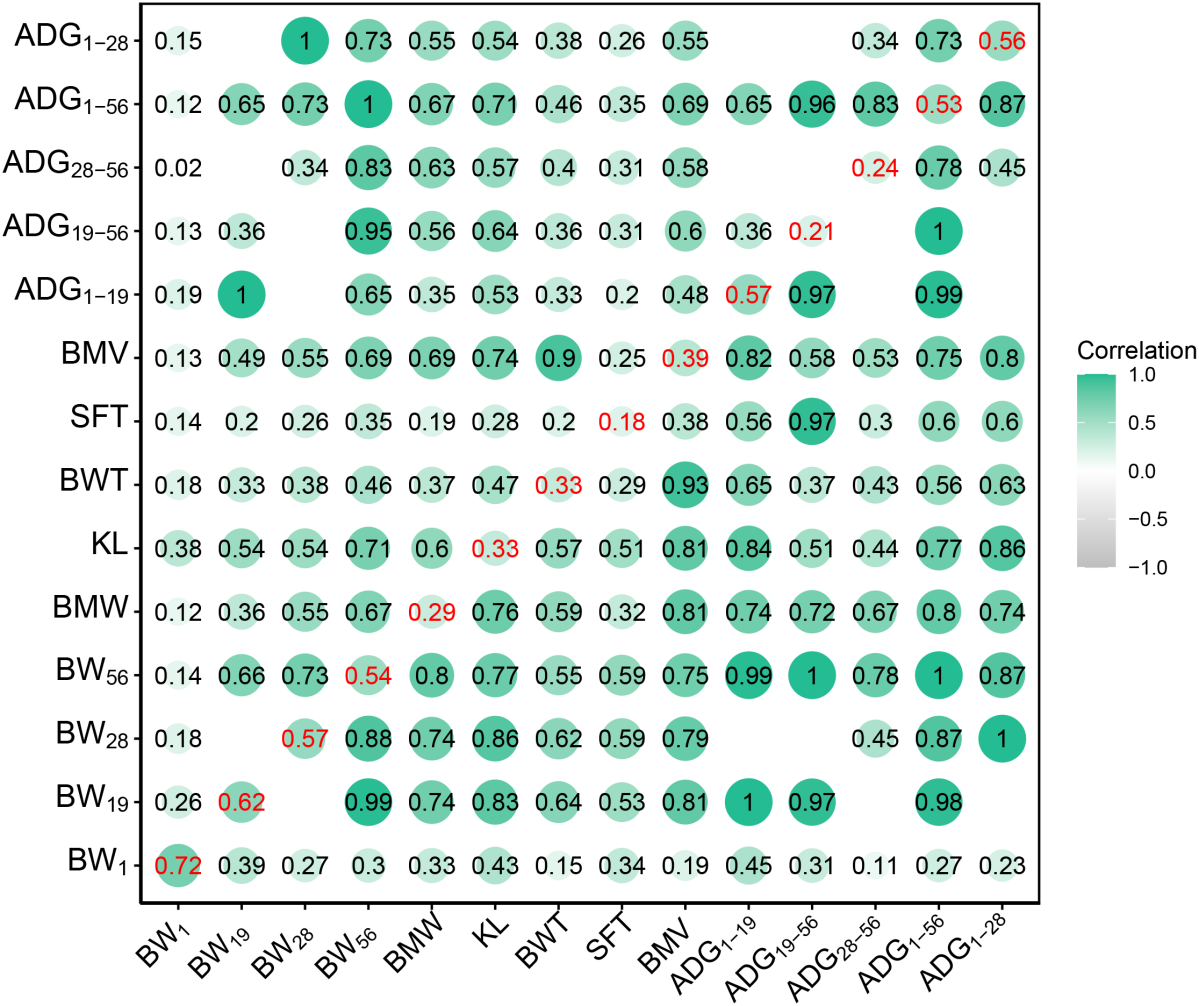
The genetic parameters of duck growth and breast morphological traits. The diagonal elements represent the heritabilities of 14 traits (ADG, average daily gain; BMT, breast muscle thickness; BMV, breast muscle volume; BMW, breast muscle width; BW, body weight; KL, keel length; SFT, skin fat thickness). The genetic correlation (below the diagonal) and phenotypic correlation (above the diagonal) between traits are shown by circles. The color of each circle represents a strongly positive correlation (green) or a weak correlation (white).

We described the phenotype of four BW traits, five ADG traits, and five BM traits in Table 1, which contained the mean, SD, and number of records for each trait. The heritability estimates and their standard error (SE) based on the pedigree BLUP model are also shown in Table 1 and Figure 1. The heritability estimates for the BW were high (0.54–0.72). The heritability values of BW decreased with age. The highest estimate was at BW_1,_ and the lowest estimate was at BW_56_. The heritabilities of ADG traits were moderate to high (0.21–0.57). We observed high heritabilities for ADG traits in the early (ADG_1–19_ and ADG_1–28_) and whole (ADG_1–56_) growth stages, while moderate heritabilities were observed for the ADG traits after 19 days (ADG_19–56_ and ADG_28–56_). The heritabilities of BM traits were moderate (0.29–0.39), except for SFT with a low heritability (0.18).

We detected both phenotypic and genetic relationships across BW, ADG, and BM traits were positive (Figure 1). Within BW traits, BW_56_ was strongly and positively correlated with BW_19_ (0.99) and BW_28_ (0.88), while the genetic and phenotypic correlations between BW1 and other BW traits were weak. As expected, all ADG traits had moderate or strong genetic correlation with BW_19_, BW_28,_ and BW_56_ (0.45–1.00), except for BW_1_ with little or moderate (0.11–0.45) genetic correlation. The genetic correlation within BM traits was dynamic, ranged from 0.29 to 0.93. The genetic correlation between BM traits and BW traits (except for BW_1_) was moderate or strong (0.55–0.86).

### 3.2 Genomic prediction using GBLUP and ssGBLUP models

**FIGURE 2:**
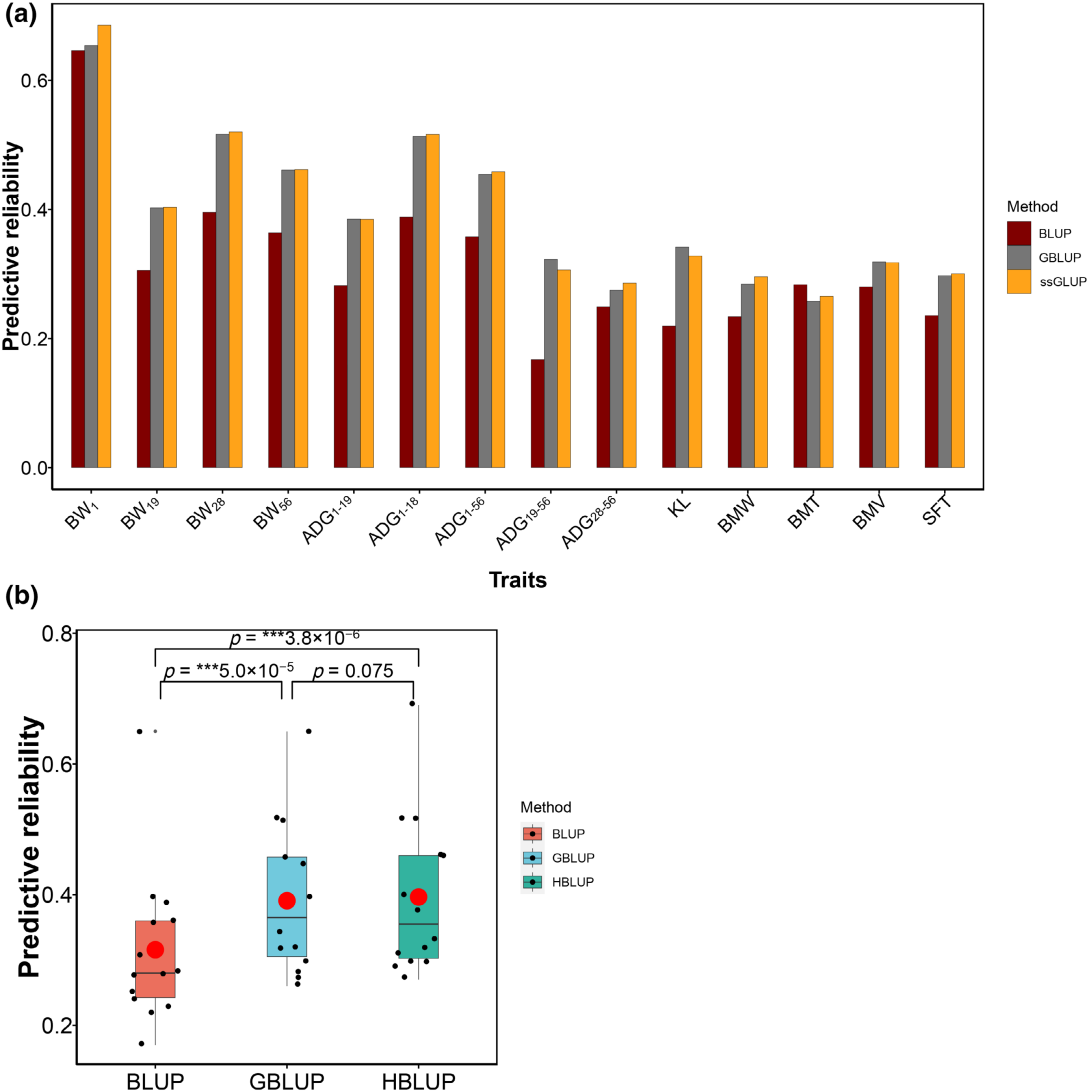
The predictive reliability of duck growth and BM traits by pedigree BLUP, GBLUP, and ssGBLUP. (a) The predictive reliability of genomic prediction for each trait using pedigree BLUP, GBLUP, and ssBLUP. The predictive reliability of pedigree BLUP, GBLUP, and ssBLUP was denoted by the dark red, gray, and orange bars, respectively. ADG, average daily gain; BMT, breast muscle thickness; BMV, breast muscle volume; BMW, breast muscle width; BW, body weight; KL, keel length; SFT, skin fat thickness. (b) The comparison of predictive reliability for pedigree BLUP, GBLUP, and ssBLUP. P values were calculated using paired *t*‐test. The red dot represents the average value of predictive reliability for each model.

Figure 2 and Table 2 present the genomic predictive reliabilities for 14 traits using pedigree BLUP, GBLUP, and ssGBLUP methods. The reliabilities of GBLUP were relatively high for BW traits (0.41–0.65), moderate for BM traits (0.26–0.33), and moderate to high for ADG traits (0.28–0.51). As expected, the prediction reliabilities using GBLUP were higher compared to those from pedigree BLUP for almost all the traits (paired *t*‐test *p* = $5.04\times 10^{-5}$), except for the BMT (Figure 2a,b). We detected the noticeable increment in predictive ability of GBLUP for ADG_19–56_ (0.16), ADG_1–28_ (0.12), BW_28_ (0.12), KL (0.12), ADG_1–19_ (0.10), BW_19_ (0.10), BW_56_ (0.10), and ADG_1–56_ (0.10). The average increment of predictive reliability between GBLUP and BLUP was 0.08 across all 14 traits. The ssGBLUP model, which combines the non‐genotyped individuals, was applied to estimate GEBV and to assess prediction reliabilities for all the BW, ADG, and BM traits. The ssGBLUP performed better than GBLUP in most traits, although the difference was not statistically significant (paired *t*‐test *p* = 0.075, Figure 2b). We observed a remarkable improvement of predictive reliability from GBLUP to ssGBLUP in BW1 trait with increment of 3.2%, while the increments of predictive reliability in other traits were relatively weak. The average increment of predictive reliability between ssGBLUP and GBLUP was 0.003 across all 14 traits.

**TABLE 2:** The predictive reliability of GS for duck growth and breast morphological traits using different models.

Category	Traits	BLUP	GBLUP	HBLUP	BayesN	BayesB	BayesCπ	BayesS	BayesR
Body weight	BW_1_	0.65	0.65	0.69	0.65	0.65	0.65	0.65	0.64
	BW_19_	0.31	0.40	0.40	0.40	0.40	0.40	0.42	0.32
	BW_28_	0.40	0.52	0.52	0.52	0.52	0.51	0.51	0.44
	BW_56_	0.36	0.46	0.46	0.56	0.53	0.46	0.47	0.45
Growth	ADG_1–19_	0.28	0.39	0.38	0.39	0.38	0.39	0.40	0.32
	ADG_1–28_	0.39	0.51	0.52	0.51	0.51	0.51	0.51	0.43
	ADG_1–56_	0.36	0.45	0.46	0.56	0.52	0.46	0.47	0.45
	ADG_19–56_	0.17	0.32	0.31	0.33	0.33	0.34	0.34	0.23
	ADG_28–56_	0.25	0.27	0.29	0.30	0.28	0.28	0.28	0.21
Breast morphological	KL	0.22	0.34	0.33	0.39	0.35	0.34	0.34	0.33
	BMW	0.23	0.28	0.30	0.38	0.36	0.29	0.29	0.25
	BMT	0.28	0.26	0.27	0.27	0.27	0.27	0.28	0.26
	BMV	0.28	0.32	0.32	0.32	0.32	0.32	0.32	0.30
	SFT	0.24	0.30	0.30	0.29	0.29	0.30	0.30	0.27

*Note*: Data showing the average predictive reliability for each trait using fivefold cross‐validation.

Abbreviations: ADG, average daily gain; BMT, breast muscle thickness; BMV, breast muscle volume; BMW, breast muscle width; BW, body weight; KL, keel length; SFT, skin fat thickness.

### 3.3 Genomic prediction using Bayesian models

**FIGURE 3:**
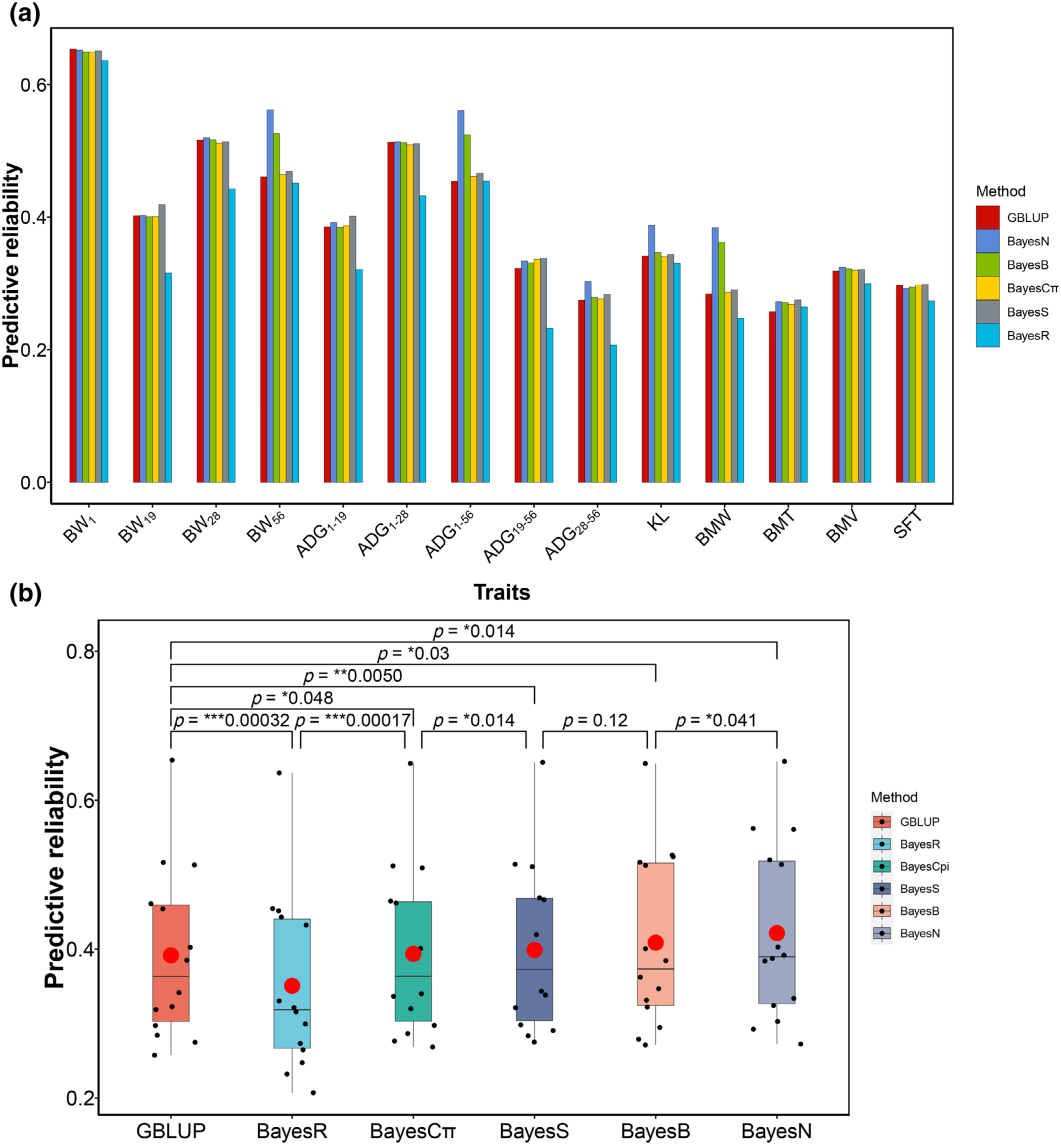
The predictive reliability of duck growth and BM traits by Bayesian models. (a) The predictive reliability of genomic prediction for each trait using GBLUP and five Bayesian models. ADG, average daily gain; BMT, breast muscle thickness; BMV, breast muscle volume; BMW, breast muscle width; BW, body weight; KL, keel length; SFT, skin fat thickness. (b) The comparison of predictive reliability between different models. *p* Values were calculated using paired *t*‐test. The red dot represents the average value of predictive reliability for each model.

The genomic predictive reliability for BW, BM, and ADG traits using Bayesian methods is summarized in Table 2. Bayesian models (except for BayesR) significantly achieved greater predictive reliability than the GBLUP model in almost all the traits, except for BW_1_ traits (Figure 3a,b, paired *t*‐test *p* < 0.05). The BayesN was the best model for increasing the predictive reliability in nine of the 14 traits, including BW_28_, BW_56_, ADG_1–28_, ADG_28–56_, ADG_1–56_, BMW, BMT, BMV, and KL. The increment in predictive reliability obtained using BayesN with respect to GBLUP was more noticeable in ADG_1–56_ (0.11), BW_56_ (0.10), and BMW (0.09). BayesN yielded an average of 3.2% higher accuracy than GBLUP (paired *t*‐test *p* = 0.014). The BayesS model, accounting for MAF and LD weights of markers, had the best reliability performance in BW_19_, ADG_1–19_, ADG_19–56_, and SFT. BayesS yielded an average of 0.7% higher accuracy than GBLUP (paired *t*‐test *p* = 0.005). BayesR had a poor performance in predictive reliability for most traits, except for BMT and ADG_1–56_. The average predictive reliability for all 14 traits was BayesN > BayesB ≈ BayesS > BayesC > GBLUP > BayesR (Figure 3b).

### 3.4 The marker density affects genomic predictions

**FIGURE 4:**
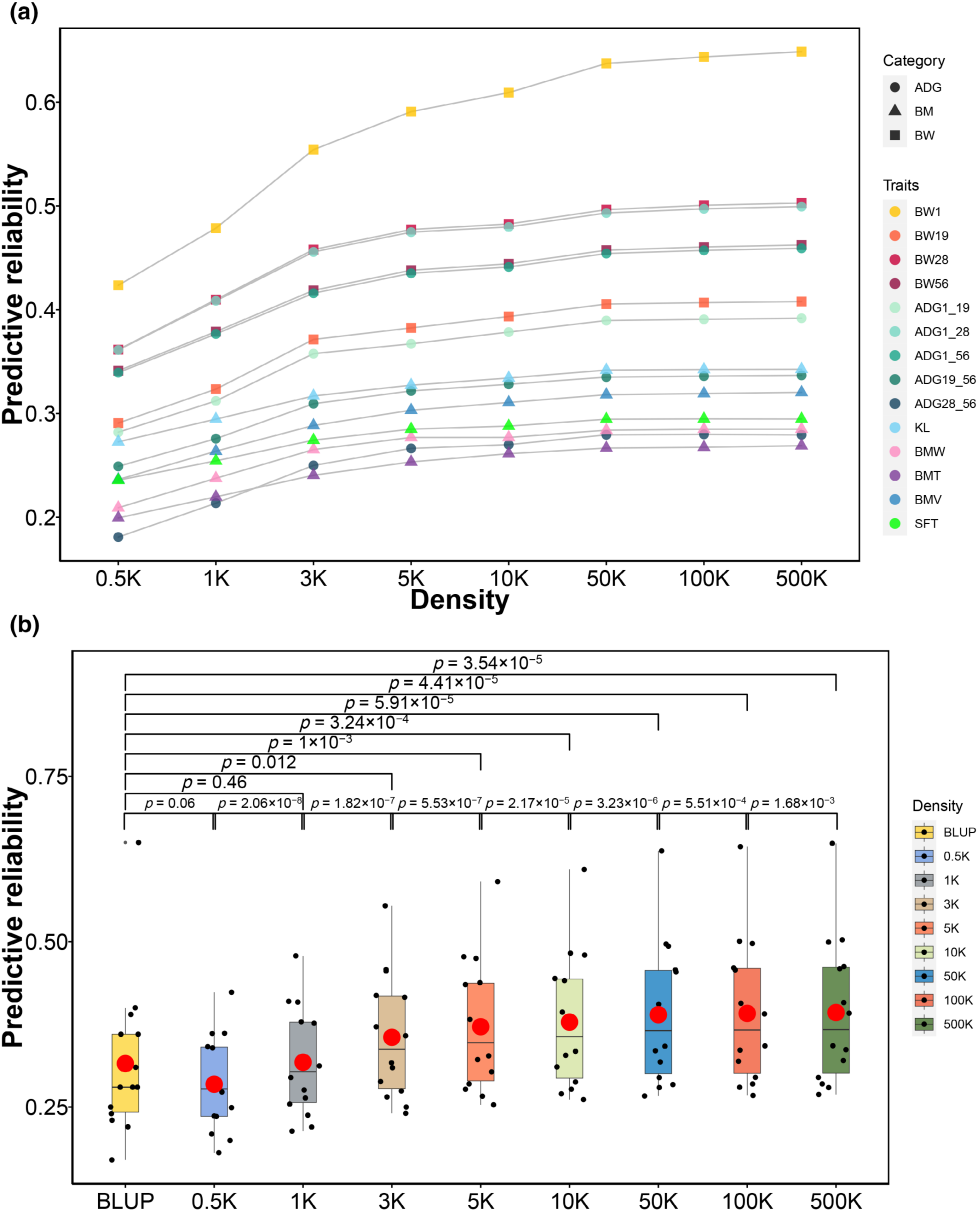
The permutation of marker density affects the predictive reliability of GS in duck growth and BM traits by GBLUP model. (a) The predictive reliability of GS changes by the various markers' density in 14 traits. The shapes circle, triangle, and square represent ADG, BM, and BW traits, respectively. ADG, average daily gain; BMT, breast muscle thickness; BMV, breast muscle volume; BMW, breast muscle width; BW, body weight; KL, keel length; SFT, skin fat thickness. (b) The comparison of predictive reliability for different marker densities. *p* Values were calculated using paired *t*‐test. The red dot represents the average value of predictive reliability for each group.

Since genotyping with medium‐ or high‐density SNP arrays is relatively expensive in poultry, we evaluated the impact of low‐density panel reduction on predictive reliability of GS. We randomly selected 0.5, 1, 3, 5, 10, 50, 100, and 500 K from the original sequencing markers. The predictive reliability for each trait was calculated by averaging the cross‐validation results of 30 random permutations. We found that the predictive reliability rapidly increased with the increase of marker density from 0.5 to 3 K and then moderately increased from 3 to 5 K (Figure 4a,b). The predictive reliability showed limited improvement when the marker density exceeded 50 K. The average predictive reliability of all traits was 0.32 for 1 K SNP markers, which exceeded the average reliability of pedigree BLUP (0.31, Table S1). The predictive capability (current reliability divided by the reliability performance of 500 K) of 50 K density reached 99.3%. The 3 K SNP markers with a predictive capability of 90.8% had a significantly better reliability performance than pedigree BLUP, which required further attention (paired *t*‐test *p* = 0.012, Figure 4b).

## 4 DISCUSSION

In this study, we estimated the genetic parameters and compared the predictive performance between GS and traditional BLUP on 14 duck growth and BM traits. We also assessed predictive performance of GS using various models and marker densities.

The heritability estimates for the body weight were high (0.54–0.72). The BW_56_ (8 weeks) heritability (0.54) was closed to previous results (0.53) by Tieshan et al. (2011), but higher than the result (0.46) shown by Tai et al. (1989). The heritability of BW_19_ (0.62) was higher than the results of BW_21_ (0.45) (Szwaczkowski et al., 2010) and BW_21_ (0.29) (Li et al., 2020) as shown by previous studies. The heritability estimates of BW traits decreased with age, and this trend was also observed in a crossbreed chicken population (Teng et al., 2019). As the value and variation of the hatch weight (BW_1_) were small, the phenotypic and genetic correlation between ADG_1–19_ and BW_19_, ADG_1–28_ and BW_28_, and ADG_1–56_ and BW_56_ was very high. The heritabilities of ADG_1–19_, ADG_1–28,_ and ADG_1–56_ were also closed to BW_19_, BW_28_, and BW_56_, respectively. The moderate heritabilities were observed for ADG_19–56_ (0.21) and ADG_19–28_ (0.24), which were lower than ADG_14–42_ (0.38) (Zhang et al., 2017) and ADG_21–42_ (0.36) (Li et al., 2020) reported by previous studies. The moderate heritabilities of BM traits at 8 weeks (0.29–0.39) were closed to another study in Pekin duck population (0.22–0.45) (Xu et al., 2011), but higher than BMW (0.19) and BMT (0.14) at 7 weeks (Szwaczkowski et al., 2010). Although BW_1_ had the highest heritability, BW_1_ had weak correlations with other traits, which implied the acquired weight and growth of ducks were limitedly determined by the congenital hatch weight. These BM traits were moderately or strongly correlated with body weight (0.55–0.86) after 19 days, which were consistent with a previous study in Pekin ducks (Kokoszyński et al., 2019). These results indicate that the traits measured by B‐ultrasound are effective in the selection of growth and weight traits in ducks.

Our results revealed that the methods with marker information generally provided higher predictive performance of GS than the traditional BLUP method; for example, GBLUP yielded 16% and 12% higher accuracies than BLUP in ADG_19–56_ and BW_28_, respectively. The prediction ability of GS on growth and BM traits increased by 8% on average. These improvements are consistent with validation results of GS in other poultry (Hidalgo et al., 2021; Liu et al., 2014; Yang et al., 2021) or livestock (Saatchi et al., 2011; Tribout et al., 2012; VanRaden et al., 2009). These results indicate that GS can be more accurate in predicting breeding values, which may be due to the fact that marker information is more accurate than pedigree in predicting the realized relationships. The high predictive ability of GS in BW traits suggests that the better performance of GS could be detected in traits with high heritability. Similar findings were obtained from additional studies where there was a significantly positive correlation between heritability and predictive ability (Clark et al., 2012; Luan et al., 2009). Overall, the ssGBLUP outperformed GBLUP in most traits in our study, as reported in the case of growth traits in chicken (Gao et al., 2019). Compared to the GBLUP method, ssGBLUP method allows for the utilization of larger datasets of phenotypic information, which helps genomic markers capture any QTL effect or polygenic effect through GEBVs (Hayes et al., 2009). These advantages of using the ssGBLUP method are herein confirmed for ducks. The advantage of ssGBLUP is increased with more non‐genotyped individuals joined (Zhao et al., 2023). There were only small improvements in predictive reliability from GBLUP to ssGBLUP for most traits (1%), which could be due to the limited number of non‐genotyped individuals being used in our study (Song et al., 2018). In our study, the heritability for BW_1_ was high (0.72), which can obtain sufficient reliability (0.65) for traditional BLUP method (Song et al., 2018). A remarkable improvement in BW_1_ traits (3%) may be due to a relatively large number of non‐genotyped individual records with sufficient heritability being used.

In general, Bayesian models outperform GBLUP when there are fewer quantitative trait loci (QTL) than independent chromosome segments, while GBLUP tends to perform better or have similar performance to Bayesian methods for traits affected by many QTLs with small effects (Chen et al., 2014; Daetwyler et al., 2010; van den Berg et al., 2015). In this study, we found Bayesian models outperformed GBLUP models in BW_56_, ADG_1–56_, ADG_19–56_, ADG_28–56_, ADG_1–56_, BMW, BMT, BMV, and KL, suggesting a limited number of major QTLs control these harvest weight and BM traits. The predictive reliability of GBLUP was better or close to Bayesian models for BW_1_, BW_19_, BW_28_, ADG_1–19_, ADG_1–28,_ and SFT, indicating the early body weight and weight gain affected by many QTLs with small effects. In most traits, the BayesN and BayesS were the most accurate models for predicting breeding values. Zeng, Garrick, et al. (2018) and Karaman et al. (2020) reported the advantages of BayesN methods. BayesS is a robust approach that has been used in various studies by Zeng, de Vlaming, et al. (2018) and Joshi et al. (2021). BayesR had a poor performance in the growth and BM traits of our population. Other studies also did not find BayesR to be more accurate than GBLUP at genomic prediction (Daetwyler et al., 2012; Pérez‐Enciso et al., 2017). The BayesR model assumes that there are four different marker distributions, which can shrink large effects heavily. This tendency tends to overperform the GBLUP approach when a small number of loci with large effects exist in traits (Daetwyler et al., 2010). Such major loci may be rare in most growth and BM traits of this study (Deng et al., 2019; Xi et al., 2023).

Duck populations have high fertility rates, which necessitate the genotyping of more ducklings when GS is conducted. This can be costly. Therefore, cost‐effective strategies need to be developed. In our study, 1 K marker density could achieve similar predictive ability as traditional BLUP breeding strategies, which indicates GS has great potential in duck breeding. When marker density is below 3 K, the predictive ability of the model dramatically increases. The predictive ability of GS has surpassed the best expected increase period after the density of 3 K, indicating that 3 K is a cost‐effective choice for duck GS application. After 50 K of marker density, further increase in marker density results in little to no additional improvement. Similar phenomena have been observed in other species (Ning et al., 2022; Wang et al., 2017), though the threshold of the plateau might be different, which might be affected by the LD of markers. When there is strong LD, fewer markers are needed to capture all segments in a population (Wientjes et al., 2013). The 50 K of the plateau suggests density marker can achieve ideal predictive ability for duck growth and BM traits.

For practical applications of GS, the sample size of the population employed in this study remains insufficient. Nevertheless, the relevant results obtained from this study hold instructive significance for subsequent GS of duck growth and BM traits. In future studies, we intend to establish a larger reference population for conducting GS research on growth traits specifically in a purebred duck breed.

## 5 CONCLUSIONS

Our findings suggested that genomic prediction had considerable improvements on predictive ability than the method based only on pedigree in duck growth and BM traits. The single‐step genetic evaluation is a feasible approach for increasing predictive ability, especially when adding more non‐genotyped individuals. BayesN and BayesS could bring noticeable improvement in the predictive ability of GS and require attention. In addition, reducing SNP density is an effective strategy to reduce the cost of GS for growth and BM traits.

## FUNDING INFORMATION

This work was supported by grants from the National Key Research and Development Project of China (2023YFD130000201), the Key Technologies Research on New Breed of Broiler Poultry by Integration of Breeding, Reproduction and Promotion (2021CXGC010805‐02), the Taishan Industry Leadership Talent Project of Shandong province in China (TSCY20190108), the China Agriculture Research System of MOF and MARA (CARS‐42), and the Science and Technology Innovation Project of the Chinese Academy of Agricultural Sciences (CXGC‐IAS‐09).

## CONFLICT OF INTEREST STATEMENT

The authors have declared no competing interests.

## Supporting information


Table S1

## Data Availability

All the genotype data have been deposited in the Sequence Read Archive (https://www.ncbi.nlm.nih.gov/sra) with the accession numbers PRJNA471401 and PRJNA450892.
